# Repeat pneumococcal polysaccharide vaccination does not impair functional immune responses among Indigenous Australians

**DOI:** 10.1038/cti.2017.46

**Published:** 2017-10-06

**Authors:** Paul V Licciardi, Edwin Hoe, Zheng Quan Toh, Anne Balloch, Sarah Moberley, Paula Binks, Rachel Marimla, Amanda Leach, Sue Skull, Kim Mulholland, Ross Andrews

**Affiliations:** 1 Pneumococcal Research Group, Murdoch Children’s Research Institute, Melbourne, Victoria, Australia; 2 Department of Paediatrics, University of Melbourne, Melbourne, Victoria, Australia; 3 Menzies School of Health Research, Child Health Division, Charles Darwin University, Darwin, Northern Territory, Australia; 4 Division of Paediatrics and Child Health, University of Western Australia, Perth, Western Australia, Australia; 5 Department of Child Health Research, Princess Margaret Hospital for Children, Perth, Western Australia, Australia; 6 London School of Hygiene and Tropical Medicine, London, UK

## Abstract

Indigenous Australians experience one of the highest rates of pneumococcal disease globally. In the Northern Territory of Australia, a unique government-funded vaccination schedule for Indigenous Australian adults comprising multiple lifetime doses of the pneumococcal polysaccharide vaccine is currently implemented. Despite this programme, rates of pneumococcal disease do not appear to be declining, with concerns raised over the potential for immune hyporesponse associated with the use of this vaccine. We undertook a study to examine the immunogenicity and immune function of a single and repeat pneumococcal polysaccharide vaccination among Indigenous adults compared to non-Indigenous adults. Our results found that immune function, as measured by opsonophagocytic and memory B-cell responses, were similar between the Indigenous groups but lower for some serotypes in comparison with the non-Indigenous group. This is the first study to document the immunogenicity following repeat 23-valent pneumococcal polysaccharide vaccine administration among Indigenous Australian adults, and reinforces the continued need for optimal pneumococcal vaccination programmes among high-risk populations.

## Introduction

Infections with *Streptococcus pneumoniae* (the pneumococcus) are a major cause of morbidity and mortality in children and adults worldwide. In Australia, Aboriginal and Torres Strait Islander peoples (hereafter respectfully referred to as ‘Indigenous’) suffer sixfold higher rates of pneumococcal disease than non-Indigenous Australians,^1^ representing one of the highest rates globally. The 23-valent pneumococcal polysaccharide vaccine (23vPPV) is currently recommended (and locally funded) for all Indigenous adolescents and adults in the Northern Territory (NT) of Australia from 15 years of age and is funded through the National Immunisation Program (NIP) for all adults in Australia aged 65 years or more, with three lifetime doses recommended (re-vaccination after 5 years and a third dose 10 years after the second, or at 50 years, whichever is later).^2^ Despite this, high rates of invasive pneumococcal disease (IPD) have persisted amongst Indigenous adults in the NT. Immune hyporesponsiveness following repeat 23vPPV immunisation has been postulated to contribute to the continued high level of susceptibility to pneumococcal disease in this population,^3^ despite having unknown clinical relevance.

## Results and Discussion

We previously described the immune response to 23vPPV among Indigenous Australians receiving first or second dose of 23vPPV and non-Indigenous Australians receiving a first dose, with a significantly lower adequate response among the Indigenous groups.^4^ While a second dose of 23vPPV appeared to further reduce serotype-specific IgG responses, this was difficult to interpret as second-dose recipients had higher pre-levels, were older and had more chronic illness (for example, diabetes).^4^ This study now further characterises the 23vPPV response, assessing opsonophagocytic responses and B-cell memory to five important serotypes (1, 6B, 14, 18C and 19A) before and after a dose of 23vPPV. The mean time since previous 23vPPV vaccination among Indigenous second-dose participants was 8 years and 9 months.

The subset of 60 participants were similar in demographics to those of the original study population (Supplementary Tables S1 and S2, online supplement). There was a higher proportion of Indigenous second-dose recipients with chronic illness (50%) compared to first-dose Indigenous and non-Indigenous groups (20% for both), consistent with the study population. Before 23vPPV, Indigenous first-dose recipients had higher IgG levels for serotypes 14 and 19A, and stronger opsonophagocytic responses (opsonophagocytic index; OI) for serotype 14, compared to non-Indigenous participants (Table 1). Compared to Indigenous first-dose recipients, Indigenous second-dose recipients had higher baseline IgG levels for serotypes 1, 6B and 14, and higher OI levels for serotype 14 (*P*<0.01 for all). The IgG responses to the remaining vaccine types were published previously.^4^

**Figure 1:**
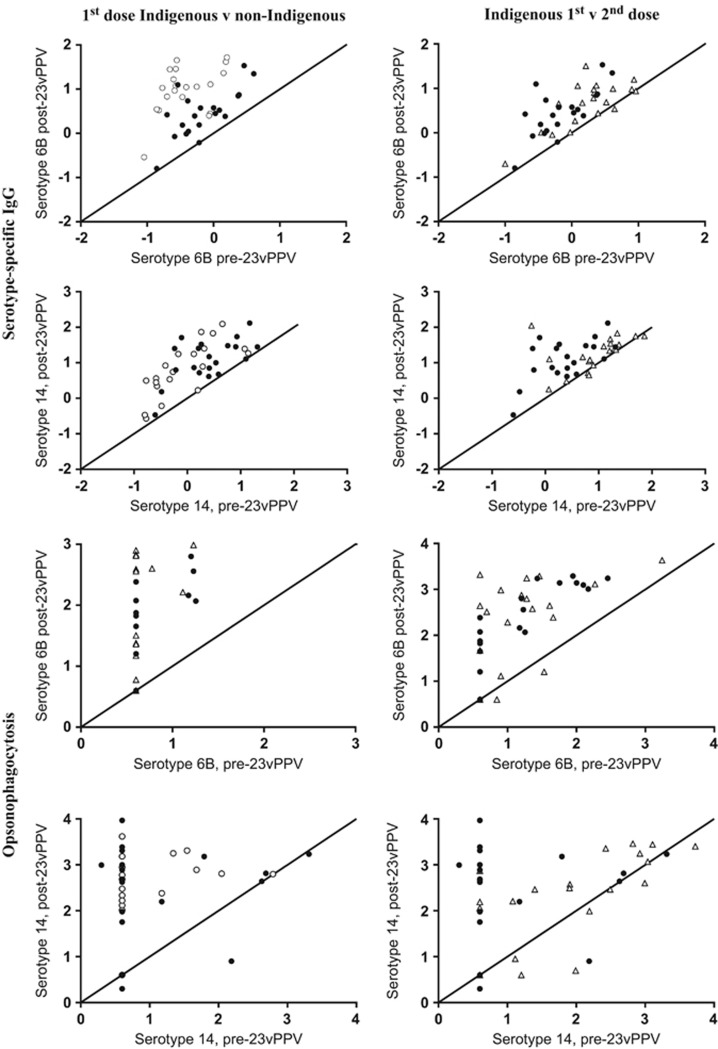
Response to 23vPPV among Indigenous and non-Indigenous Australians. The change in serotype-specific IgG (μg ml^−1^) and OI levels after 23vPPV immunisation is shown for serotypes 6B and 14. Data are log-transformed. The diagonal line represents no change from pre-23vPPV (baseline) levels. Indigenous Australians given a single dose of 23vPPV are represented by solid circles; Indigenous Australians given a second dose of 23vPPV are represented by open triangles and non-Indigenous Australians are represented by open circles.

The additional analyses undertaken on these participants found no significant differences in post-vaccination serotype-specific IgG or OI levels between the first- and second-dose Indigenous groups, whereas non-Indigenous recipients had significantly higher IgG and OI levels for serotype 1 only (*P*<0.01; Table 1). Analysis of memory B-cell numbers across all groups and at both time points revealed similar numbers, except for serotype 6B, which was higher post vaccination in non-Indigenous compared to Indigenous recipients (*P*<0.01). Regardless of baseline, IgG and opsonophagocytic assay (OPA) responses following 23vPPV increased in all groups for most serotypes examined (Figure 1 for serotypes 6B and 14; remaining serotypes shown in Supplementary Figures S1 and 2, online supplement). However, for serotype 14, Indigenous second-dose recipients responded with lower OI but not IgG than first-dose recipients.

Overall, our data suggest that while Indigenous participants do not respond as well to selected serotypes in 23vPPV compared to non-Indigenous participants, repeat 23vPPV immunisation 8–9 years after the first dose among Indigenous participants does not appear to further reduce vaccine responsiveness. It is possible that immune hyporesponsiveness may have been missed due to the small numbers or that it may occur if the doses are given more closely together. Poorer responses to 23vPPV may be due to prior carriage of pneumococci, chronic illness, environmental factors or ethnicity.^5, 6, 7, 8, 9^ However, the carriage rate was low in this study (data not shown), similar to a previous report in the population,^10^ suggesting other factors may be important in the response to 23vPPV. There remains controversy over the use of 23vPPV and its potential to induce immune hyporesponsiveness.^3^ Indeed, studies that have demonstrated hyporesponsiveness following 23vPPV have shown that this is unable to be restored by subsequent pneumococcal conjugate vaccine (PCV) use.^11, 12^ We have previously demonstrated this phenomenon in a cohort of Fijian infants given 23vPPV at 12 months of age^13^ but we also showed that this resolved after 5 years.^14^ Although memory B-cell depletion has been suggested as an explanation for this effect,^15^ this is not supported by our data. In older adults, data from several studies have shown inconsistent findings in relation to repeated 23vPPV immunisation due to differences in study design, populations studied or assays used.^9, 16, 17^ We have previously reported that the use of adequate response criteria (as per the American Academy of Allergy, Asthma and Immunology) reveals differences in responsiveness that may not be apparent when directly comparing geometric mean levels of antibody.^4^ Adapting these criteria for opsonophagocytic responses may reveal potential differences and is worth further consideration. The lack of validated response criteria for OPA or memory B-cell analysis may be one explanation why hyporesponsiveness was not observed in this study compared to our previous report based on serotype-specific IgG response criteria.

Whilst our study is the first to compare functional responsiveness to 23vPPV among Indigenous and non-Indigenous Australians and uses the most up-to-date methods available, we acknowledge the limitations of our design, including the small sample size and the inability to adjust for confounders such as comorbidities. Furthermore, while the serotypes selected in this study represent common disease-causing serotypes, it is possible that suboptimal responses to other vaccine serotypes were missed. Assessment of opsonophagocytosis and B-cell memory to all 23 serotypes, while challenging, could provide a more complete understanding of 23vPPV responsiveness.

Implementation of rigorous and optimal pneumococcal vaccination programmes for high-risk populations is critical to ensure the continued reduction in IPD in these settings. In consideration of the 13-valent PCV (PCV13) use in adults as part of the NIP in Australia, the use of 23vPPV is currently under review by the Australian Pharmaceutical Benefits Advisory Committee. PCV13 could therefore be a strategy to enhance protection in adults in high-risk communities such as Indigenous Australians.

## Methods

### Study population

The details of the study design has been published previously.^4^ Briefly, Indigenous Australians were recruited into the study based on whether they were due to receive a first or second dose of 23vPPV, as well as a non-indigenous Australian control group. A subset of 20 participants in each of these three groups were analysed in this current study. Blood samples were collected before and 28 days after a dose of 23vPPV. None of the participants had received PCV previously. Ethical approval for this study was obtained from the Human Research Ethics Committee of the NT Department of Health and the Menzies School of Health Research (06/57), including assessment by the Aboriginal Ethics Sub-Committee. All investigators were blinded for the laboratory outcomes.

### Pneumococcal serotype-specific IgG responses

Measurement of serotype-specific IgG responses to all 23vPPV serotypes was undertaken using a modified World Health Organisation-recommended enzyme-linked immunosorbent assay^18^ and previously reported. Responses to serotypes 1, 6B, 14, 18C and 19A in this subset are reported in this manuscript.

### Opsonophagocytic responses

A multiplexed OPA was used to measure the functional antibody response for serotypes 6B, 14, 18C and 19A, and a singleplex assay used for serotype 1 according to a previously published method from our laboratory.^14^

### Memory B-cell responses

Pneumococcal-specific memory B-cell numbers were enumerated by Elispot using an established method published by our laboratory.^14^ Briefly, peripheral blood mononuclear cells (PBMCs) were isolated from heparinised whole blood and cultured for 6 days with *Staphylococcus aureus* Cowan strain—Pansorbin cells (1:5000), 2.5 μg ml^−1^ CpG and 83 ng ml^−1^ pokeweed mitogen. After 6 days, PBMCs were collected, washed and plated onto Elispot plates coated with serotypes 1, 6B, 14, 18C and 19A.

### Statistical analysis

Serotype-specific IgG and OIs were presented as geometric mean concentrations or geometric mean OIs with 95% confidence intervals, respectively. Log-transformed data were used for statistical analyses of IgG and OPA data by using paired Student’s *t*-tests. Means and standard error for memory B cells were analysed using unpaired Student’s *t*-tests. *P*-values <0.05 were considered significant.

## Figures and Tables

**Table 1:** Immune response to 23vPPV among Indigenous Australians receiving a first or second dose compared to first-dose non-Indigenous Australians

	*Indigenous first dose (*N*=20)*		*Indigenous second dose (*N*=20)*		*Non-Indigenous first dose (*N*=20)*	
*Outcome*	*Pre*	*Post*	*Pre*	*Post*	*Pre*	*Post*
*Serotype-specific IgG (μg ml^−1^)*^a^						
1	0.54 (0.37–0.77)	2.45 (1.29–4.63)	1.43** (0.93–2.20)	3.41 (2.03–5.71)	0.38 (0.25–0.56)	9.78** (5.49–17.42)
6B	0.70 (0.45–1.09)	2.85 (1.59–5.13)	1.76** (1.04–2.97)	4.44 (2.53–7.79)	0.44 (0.30–0.64)	2.87 (1.67–4.96)
14	2.43 (1.35–4.37)	12.3 (6.51–23.26)	8.71** (4.83–15.69)	17.98 (10.74–30.09)	0.87* (0.46–17.46)	6.51 (2.91–14.59)
18C	0.80 (0.44–1.46)	3.72 (2.04–6.80)	1.35 (0.84–2.18)	4.05 (2.55–6.44)	0.58 (0.35–0.98)	7.77* (4.94–12.22)
19A	8.11 (5.2–12.64)	10.65 (6.51–17.44)	6.73 (4.46–27.65)	10.16 (7.01–14.71)	2.61*** (1.80–3.78)	6.83 (4.28–10.90)
						
*Opsonophagocytosis (OI)*^b^						
1	5 (3–6)	15 (6–39)	5 (4–7)	14 (6–32)	4 (4–4)	240 (88–655)***
6B	16 (8–33)	181 (70–468)	16 (8–33)	202 (70–583)	6 (4–9)*	131 (39–436)
14	13 (5–33)	244 (79–751)	86 (30–251)**	218 (78–610)	9 (5–17)	205 (72–581)
18C	16 (8–35)	83 (30–231)	14 (7–28)	112 (43–290)	6 (4–9)*	191 (67–545)
19A	14 (7–29)	62 (24–160)	22 (9–51)	95 (37–240)	9 (5–18)	108 (41–288)
						
*Memory B cell ( × 10*^*6*^ *PBMC per ml)*^c^						
1	4.1 (1.7)	3.8 (1.3)	3.0 (1.0)	2.6 (0.8)	1.4 (0.9)	4.6 (2.0)
6B	2.7 (0.9)	1.7 (0.5)	2.1 (0.6)	1.2 (0.4)	1.8 (0.6)	4.6 (0.9)**
14	1.9 (0.9)	2.3 (1.0)	2.3 (0.7)	1.3 (0.5)	1.0 (0.3)	3.2 (1.1)
18C	4.3 (1.6)	4.1 (1.7)	3.8 (1.8)	2.1 (0.6)	1.6 (0.5)	2.8 (0.7)
19A	3.5 (1.5)	1.7 (0.6)	4.0 (1.2)	1.7 (0.4)	1.7 (0.5)	2.9 (0.7)

Abbreviations: OI, opsonophagocytic index; PBMC, peripheral blood mononuclear cell; 23vPPV, 23-valent pneumococcal polysaccharide vaccine.

*P<0.05, **P<0.01, ***P<0.001 compared to Indigenous first-dose group.

a Results expressed as geometric mean concentration (±95% confidence intervals).

b Results expressed as geometric mean opsonophagocytic index (±95% confidence intervals).

c Results expressed as mean (±s.e.m.). Serotype-specific IgG responses to the remaining serotypes in 23vPPV have been previously reported.^4^
