# Supplementary material for: Repeat pneumococcal polysaccharide vaccination does not impair functional immune responses among Indigenous Australians
Source: Clin Transl Immunology. 2017 Oct 6;6(10):e158–. doi: 10.1038/cti.2017.46 (PMC5671990; doi:10.1038/cti.2017.46)
Supplement: Supplementary Tables [file cti201746x1.docx]

Table S1. Demographic characteristics of study participants

|  | Indigenous  1^st^ dose (n=20) | Indigenous  2^nd^ dose (n=20) | Non-Indigenous  1^st^ dose (n=20) |
| --- | --- | --- | --- |
| Days post-vaccination Mean (range) | 35 (25 to 97) | 28 (23 to 35) | 29 (25 to 35) |
| Age Mean (range) | 35 (16 to 54) | 38 (19 to 59) | 31 (16 to 40) |
| Proportion Male | 10 (50%) | 17 (85%)* | 7 (35%) |
| Chronic illness^†^ | 3 (20%) | 10 (50%)* | 3 (20%) |

*p=0.04 compared to Indigenous first dose recipients; ^†^e.g. diabetes

Table S2: Number of participants with an adequate response to 23vPPV

|  | **Adequate response*** | |
| --- | --- | --- |
| *Group* | *≥12 serotypes (%)* | *Median number of serotypes* |
| Indigenous 1^st^ dose (n=20) | 17 (85) | 17 |
| Indigenous 2^nd^ dose (n=20) | 16 (80) | 17 |
| Non-Indigenous 1^st^ dose (n=20) | 20 (100) | 21 |

*defined *a priori* according to Moberley et al^4^
