# Supplementary material for: Repeat pneumococcal polysaccharide vaccination does not impair functional immune responses among Indigenous Australians
Source: Clin Transl Immunology. 2017 Oct 6;6(10):e158–. doi: 10.1038/cti.2017.46 (PMC5671990; doi:10.1038/cti.2017.46)
Supplement: Supplementary Figures [file cti201746x2.docx]

**Indigenous 1^st^ v 2^nd^ dose**

**1^st^ dose Indigenous v non-Indigenous**

Figure S1: Response to 23vPPV among Indigenous and non-Indigenous Australians. The change in serotype-specific IgG (µg/mL) levels after 23vPPV immunisation is shown for serotypes 1, 18C and 19A. Data is log-transformed. The diagonal line represents no change from pre-23vPPV (baseline) levels. Indigenous Australians given a single dose of 23vPPV are represented by solid circles; Indigenous Australians given a second dose of 23vPPV are represented by open triangles; non-Indigenous Australians are represented by open circles.

**Indigenous 1^st^ v 2^nd^ dose**

**1^st^ dose Indigenous v non-Indigenous**

Figure S2: Response to 23vPPV among Indigenous and non-Indigenous Australians. The change in opsonophagocytic index (OI) levels after 23vPPV immunisation is shown for serotypes 1, 18C and 19A. Data is log-transformed. The diagonal line represents no change from pre-23vPPV (baseline) levels. Indigenous Australians given a single dose of 23vPPV are represented by solid circles; Indigenous Australians given a second dose of 23vPPV are represented by open triangles and non-Indigenous Australians are represented by open circles.
